# Evaluating Quality of Decision-Making Processes in Medicines' Development, Regulatory Review, and Health Technology Assessment: A Systematic Review of the Literature

**DOI:** 10.3389/fphar.2017.00189

**Published:** 2017-04-10

**Authors:** Magdalena Bujar, Neil McAuslane, Stuart R. Walker, Sam Salek

**Affiliations:** ^1^Centre for Innovation in Regulatory Science (CIRS)London, UK; ^2^Department of Pharmacy, Pharmacology and Postgraduate Medicine, School of Life and Medical Sciences, University of HertfordshireHatfield, UK; ^3^Institute for Medicines DevelopmentCardiff, UK

**Keywords:** measurement instrument, quality decision making, QDMPs, R&D, pharmaceutical, regulatory, health technology assessment

## Abstract

**Introduction:** Although pharmaceutical companies, regulatory authorities, and health technology assessment (HTA) agencies have been increasingly using decision-making frameworks, it is not certain whether these enable better quality decision making. This could be addressed by formally evaluating the quality of decision-making process within those organizations. The aim of this literature review was to identify current techniques (tools, questionnaires, surveys, and studies) for measuring the quality of the decision-making process across the three stakeholders.

**Methods:** Using MEDLINE, Web of Knowledge, and other Internet-based search engines, a literature review was performed to systematically identify techniques for assessing quality of decision making in medicines development, regulatory review, and HTA. A structured search was applied using key words and a secondary review was carried out. In addition, the measurement properties of each technique were assessed and compared. Ten Quality Decision-Making Practices (QDMPs) developed previously were then used as a framework for the evaluation of techniques identified in the review. Due to the variation in studies identified, meta-analysis was inappropriate.

**Results:** This review identified 13 techniques, where 7 were developed specifically to assess decision making in medicines' development, regulatory review, or HTA; 2 examined corporate decision making, and 4 general decision making. Regarding how closely each technique conformed to the 10 QDMPs, the 13 techniques assessed a median of 6 QDMPs, with a mode of 3 QDMPs. Only 2 techniques evaluated all 10 QDMPs, namely the Organizational IQ and the Quality of Decision Making Orientation Scheme (QoDoS), of which only one technique, QoDoS could be applied to assess decision making of both individuals and organizations, and it possessed generalizability to capture issues relevant to companies as well as regulatory authorities.

**Conclusion:** This review confirmed a general paucity of research in this area, particularly regarding the development and systematic application of techniques for evaluating quality decision making, with no consensus around a gold standard. This review has identified QoDoS as the most promising available technique for assessing decision making in the lifecycle of medicines and the next steps would be to further test its validity, sensitivity, and reliability.

## Introduction

The literature on decision making stretches back over several centuries and encompasses a wide range of academic disciplines—from philosophy and history to mathematics (Buchanan and O'Connell, 2006). More recently, the science and art of decision making have been established regarding the psychology of judgment, decision-making styles, as well as behavioral economics to enable quality decision making (Thaler and Sustein, 2009; Lovallo and Sibony, 2010; Kahneman, 2011). According to this research, one of the most fundamental distinctions in decision making is between the quality of the process and the quality of the outcome, as good decisions can be made but may lead to an unfavorable outcome due to uncertainty. Consequently, the two should be evaluated separately and the quality of the decision-making process will be the focus of this review.

Although quality is difficult to define due to its subjective nature, it is nevertheless possible to identify the elements of a quality decision-making process. Indeed, the general principles and steps for making a quality decision have been characterized by a number of academic and consultancy groups (Matheson and Matheson, 1998; Hammond et al., 1999; Blenko et al., 2010; SDG, 2011) and include identifying the problem and objectives; having creative implementable options; obtaining meaningful, reliable information upon which to base a decision; identifying clear consequences and trade-offs for each supportive element; considering uncertainty and eliminating biases; using logically correct reasoning; and making a commitment to action. More recently, these principles have been applied across a number of disciplines such as economics, environmental protection, clinical practice, nuclear safety, and government affairs, to facilitate quality decision making (Ratliff et al., 1999; Dowding and Thompson, 2003; Morton et al., 2009; Thaler and Sustein, 2009; Wagner, 2013).

However, research on decision making to enable a quality process during medicines' development, regulatory review, and health technology assessment (HTA) is less well-articulated and it is not certain how it is being applied by organizations and individuals in companies and agencies. This may be because there is limited awareness regarding the science of decision making in this area, as well as limited training and education (Bujar et al., 2016a).

Nevertheless, regulatory authorities, HTA agencies, and pharmaceutical companies have been using a number of frameworks for specific decision-making processes in addition to legislative frameworks that govern organizations. In particular, the area of benefit-risk assessment has brought certain concepts in decision making to the forefront through the usage of qualitative and quantitative tools by pharmaceutical companies and regulatory authorities (Guo et al., 2010; EMA, 2011; FDA, 2013; Tafuri, 2013; Leong et al., 2015; Pignatti et al., 2015), as well as in the area of HTA regarding inclusion of multiple decision criteria (Cole et al., 2016) and a structured assessment of comparative added benefit of a technology against the cost of treatment (Cherny et al., 2015; Schnipper et al., 2015). The second key area that has benefitted from more structured decision making is portfolio management, where companies have been using frameworks (Sharpe and Keelin, 1998; Cook et al., 2014) as well as quantitative methods and algorithms (Hassanzadeh et al., 2011; Jekunen, 2014) in order to analyze and optimize the portfolio of medicines and ultimately avoid late terminations in phase III development. The third area has been around the use of good submission and review practices by pharmaceutical companies and regulatory authorities, respectively (WHO, 2015), as well as in HTA agencies to standardize evidence generation (EUnetHTA, 2016) and to analyze the various decision-making systems for the assessment of health technologies (Rogowski et al., 2008). Finally, pharmaceutical companies, regulatory authorities, and HTA agencies have developed specific frameworks and guidelines to formalize the decision-making process of various committees (EMA, 2007; FDA, 2008; Hassanzadeh et al., 2011; CADTH, 2012).

**Figure 1:**
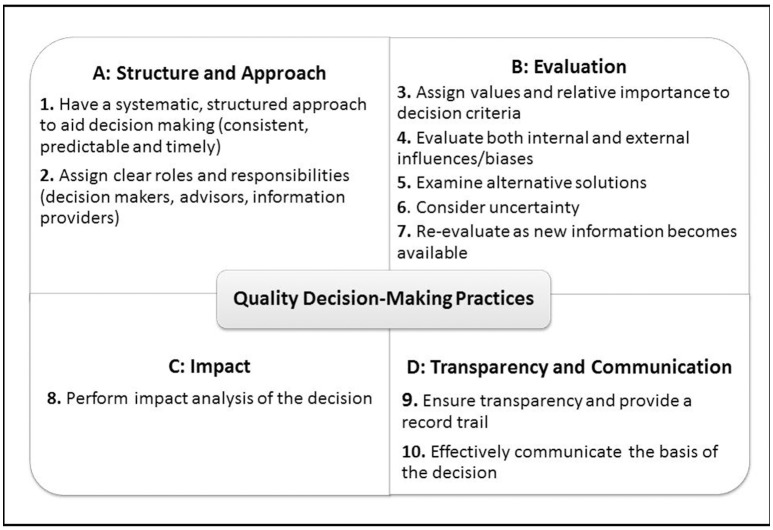
**The 10 Quality Decision-Making Practices (QDMPs)**.

Although these frameworks serve their purpose and describe the specific process steps and principles regarding decision making during development, review and HTA assessment of medicines, they do not often account for the subjective elements, such as behaviors and influences that affect the process with which individuals and organizations arrive at the final decision. In order to address this gap, a previous review of recent research on decision making has resulted in the development of 10 Quality Decision-Making Practices (QDMPs) to enable quality decision making. These were developed based on outcomes of semi-structured interviews with 29 key opinion leaders from regulatory authorities and pharmaceutical companies to investigate and identify the important issues that influence quality decision making (Donelan et al., 2015) and were considered as relevant to those two stakeholders (Bujar et al., 2016a). Moreover, the key frameworks used during medicines development, particularly in the area of benefit-risk assessment (Leong et al., 2015), as well as the science of decision making (Matheson and Matheson, 1998; Hammond et al., 1999; Blenko et al., 2010; SDG, 2011) are underpinned by this set of holistic practices. The 10 QDMPs are organized into four areas: “Structure and Approach,” “Evaluation,” “Impact,” and “Transparency and Communication” (Figure 1; Bujar et al., 2016b).

As the use of frameworks by regulatory authorities, HTA agencies and companies increases, questions remain about whether these frameworks enable better quality decision making. This could be addressed by formally evaluating the decision-making process as well as the internal and external challenges within organizations and across individuals. In the absence of other validated criteria for evaluating quality decision making in medicines development, review and HTA, the 10 QDMPs were then used as a framework for the evaluation of the quality and generalizability of techniques identified in the review. As a result, this literature review aimed to identify current techniques, including tools, questionnaires, surveys as well as studies that measure the quality of the decision-making process within regulatory authorities, HTA agencies and pharmaceutical companies. The objectives were to compare the existing techniques, assess their measurement properties, identify research gaps and recommend the way forward. Of interest would be to find a technique that is applicable to all three stakeholders in order to have a common platform for discussing, sharing and comparing issues in quality decision making throughout the lifecycle of medicines.

## Methods

A systematic review of the literature was undertaken to identify current approaches for assessing quality decision making in medicines development, regulatory review, and HTA.

### Data sources

The following databases were searched: MEDLINE (using PubMed), Web of Knowledge, Google Scholar, and Open Access theses and dissertations. Gray literature was also searched using Google. This review was limited to English-language articles and covered the 20-year period from 1996 to March 2016, which reflects the proliferation of publications in this area.

### Search terms

Initially, an exploratory search was undertaken using basic terms as key words including quality, decision making, techniques, instruments, tools, measurement, regulatory review, medicines development, and HTA. These were also used to search gray literature. The following structured search terms were constructed using PubMed guidelines and MESH terms and these were used in database searches (Decision^*^ OR “decision mak^*^” OR preference^*^) AND (“health technology” OR HTA OR reimbursement OR coverage OR regulat^*^ *OR R*&D OR “research and development” OR development OR medicine^*^) AND (agency OR committee OR assessor^*^ OR reviewer^*^ “pharmaceutical compan^*^” OR industry) AND (measur^*^ or metric^*^ OR evaluat^*^ OR assess^*^ OR apprais^*^ OR analys^*^) AND (technique OR checklist OR tool OR scale OR feedback OR survey OR questionnaire OR instrument).

### Selection procedure

The titles and abstracts resulting from this search were screened for relevance and duplication. The full text studies were obtained for all titles/abstracts that appeared to meet the inclusion/exclusion criteria or where there was any uncertainty. The full text articles were then screened and literature was also obtained by checking the references of the included articles as well as by searching the gray literature.

### Selection criteria

Included were: (1) All articles which identified a technique (tool, instrument, or questionnaire) for evaluating quality of decision making; (2) Techniques applicable to the area of medicines' development, regulatory, or HTA; (3) Techniques evaluating the decision, the decision-making process or key aspect(s) of the process and associated preferences, influences, and behaviors; (4) Studies that assess the performance of the technique by evaluating hypothetical or real (historical) decisions, vignettes, or a reflection of individual style or approach.

Excluded were (1) General discussions on decision making and quality within the area of medicines' development, review, and HTA; (2) techniques for measuring quality of decision making used specifically in disciplines other than medicines' development, regulatory review, and HTA; (3) Frameworks for structuring and documenting decision-making processes and for enabling quality to be built into decision making.

### Data extraction and synthesis

The following extracted information were recorded: title of the technique, decision area (e.g., regulatory advisory committee or medicines R&D), study subject (e.g., regulatory authority, HTA agency or industry), subject type (organizations or individuals) and method.

### Assessment of the techniques

In the absence of an alternative evaluation criteria system that captures issues relevant to the areas of medicines' development, review, and HTA assessment, the 10 QDMPs (Table 1) were used to evaluate the techniques identified in this review to ensure that each technique is evaluating all key aspects of quality decision making. The 10 QDMPs were developed based on results from semi-structured interview with 29 key opinion leaders from regulatory authorities and pharmaceutical companies to investigate and identify the important issues that influence decision making (Donelan et al., 2015). In addition, the key decision-making frameworks (Hammond et al., 1999; Blenko et al., 2010; SDG, 2011) as well as benefit-risk assessment methodologies (Leong et al., 2015; Pignatti et al., 2015) are also underpinned by these practices. In a subsequent review, these QDMPs were presented to major pharmaceutical companies and regulatory authorities, and were considered as appropriate and relevant (Bujar et al., 2016a).

**Table 1:** **Description of the 10 QDMPs**.

**QDMP**	**Description**
1. Have a systematic, structured approach to aid decision making (consistent, predictable, and timely)	Establish the decision context, objectives and assumptions made. Employ frameworks, guidelines and tools for structuring the decision-making process. Such an approach should ensure that the process is systematic, which in turn would enable better consistency compared with similar past decisions, as well as predictability and timeliness.
2. Assign clear roles and responsibilities (decision makers, advisors, information providers)	The roles and responsibilities should be clearly defined in terms of individuals who provide information (including external input), compared with those who advise on the decision or make the final decision. The roles and responsibilities of each stakeholder (regulatory authorities, HTA agencies and companies) should be transparent and well communicated, which should help manage expectations.
3. Assign values and relative importance to decision criteria	The relevant criteria for the decision must be determined to ensure that these are in line with the decision context and overall objective. The criteria should be weighted, for example, by ranking or rating their relative importance.
4. Evaluate both internal and external influences/biases	Stakeholders need to be aware of personal considerations, subjective influences and biases, acknowledge them and minimize where possible. Potential biases that need to be considered (Lovallo and Sibony, 2010): ◦ Action-oriented bias: excessive optimism, overconfidence in own judgment and gut-feeling ◦ Interest-oriented bias: inappropriate attachments and misaligned incentives. ◦ Pattern recognition: generalizing based on recent events and seeking out information that supports a favored decision, which could lead to perpetuating previous mistakes. ◦ Stability bias: preference for status quo and tendency for inertia in the presence of uncertainty.
5. Examine alternative solutions	Decision makers should actively explore possible options during the decision-making process. The alternatives need to be assessed, for example using a SWOT analysis, against the relevant decision criteria in order to determine the best outcome.
6. Consider uncertainty	The extent and limitations of available information need to be judged for each decision criterion in relation to the alternative options. Stakeholders must be explicit regarding acceptability of benefits and harms and how this affects their approach.
7. Re-evaluate as new information becomes available	This should be actively carried out at all stages during the lifecycle of medicines' development. This may be a safeguard against plunging in or procrastination and/or perpetuating previous mistakes as well as identifying cultural/organizational/hierarchical influences (e.g., individual vs. organizational, group successes and group failures).
8. Perform impact analysis of the decision	The impact of the decision needs to be considered on both internal and external stakeholders. The analysis must relate to present situation, but also to the future and should take into account elements of quality/validity of data, political/financial/competitor influences and procedures for similar decisions.
9. Ensure transparency and provide a record trail	It must be clear how the decision was made and details must be consistently documented in a manner that can be easily followed or audited by appropriate stakeholders.
10. Effectively communicate the basis of the decision	The basis of the decision needs to be appropriately communicated to the relevant stakeholders, both internally and externally.

In addition, the measurement properties of each technique were assessed in terms of

- Theoretical underpinning (development technique was based on a well-described methodological framework);
- Psychometric properties (development of technique involved psychometric tests; content validity, and internal consistency);
- Psychometric evaluations (the validity, reliability, and sensitivity of the tool was demonstrated);
- Demonstrated practicality (the technique was applied to target population through pilot studies);
- Generalizability (the technique can be used across industry, regulatory and HTA); and
- Applicability (the technique is applicable to evaluating individuals and organizations), which were considered as the key properties that need to be considered when evaluating such instruments (McDowell, 2006; Streiner et al., 2015).

### Secondary review

An independent secondary reviewer (JW, see Acknowledgments) was involved in the development of the search strategy and selection criteria, as well as article selection and data extraction. Secondary screening was carried out as follows: MB selected at random 25% of the full text papers (10 out of 38), which were re-assessed for inclusion/exclusion by the secondary reviewer against the criteria. Disagreements were resolved through discussion, and MB and JW disagreed regarding the inclusion of one paper. This was resolved by refining the inclusion criteria to make them more specific. Following this modification, 100% concordance was reached regarding the included papers. JW also independently carried out data extraction and a small number of disagreements were resolved through discussions until full agreement was reached.

## Results

For the purpose of clarity, the key results are presented in three parts:

- Part 1: Selected articles for review
- Part 2: Identified techniques for evaluating quality of decision making
- Part 3: Measurement properties of the techniques.

### Part 1: selected articles for review

**Figure 2:**
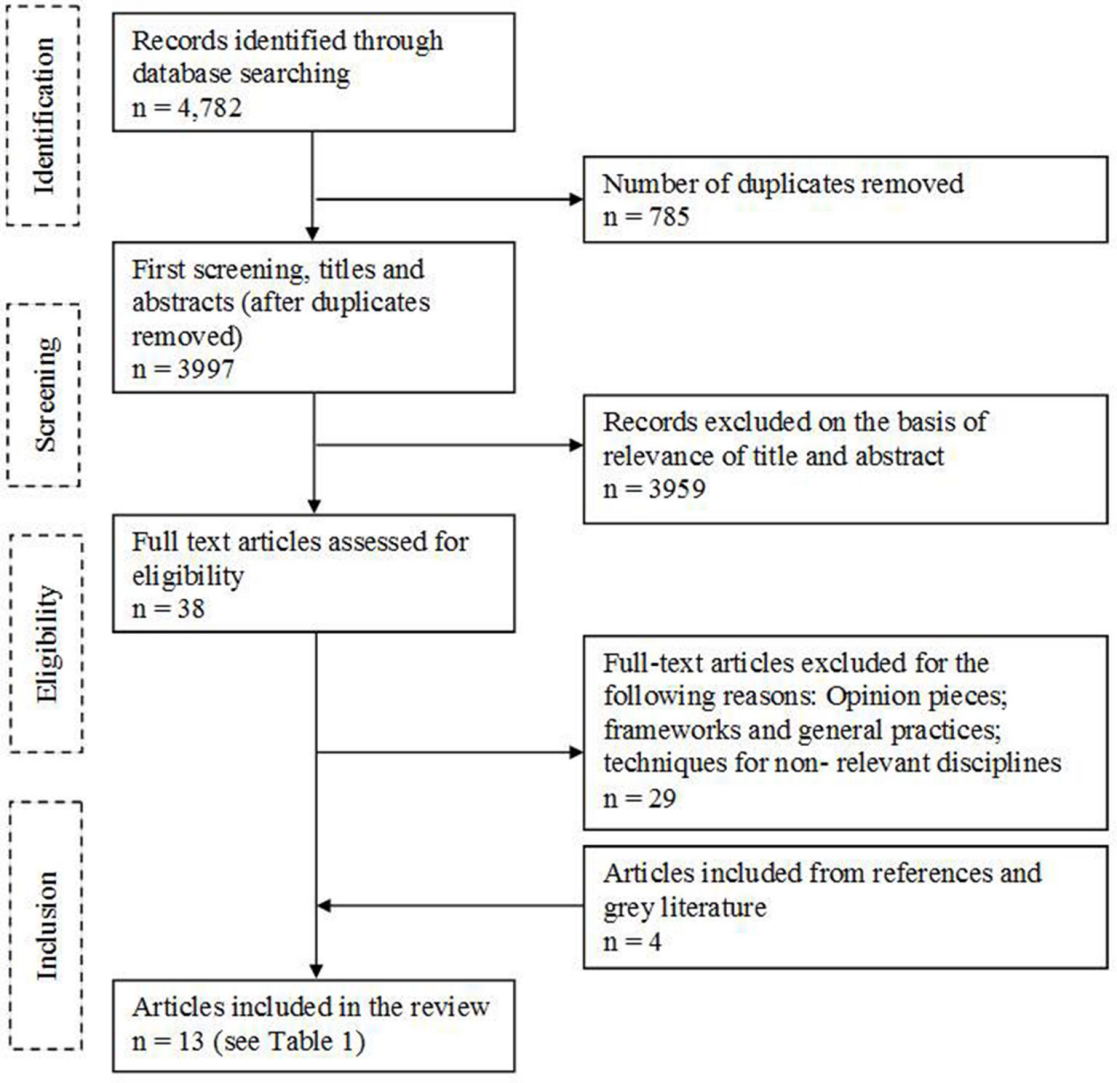
**Flow diagram of article selection**.

Of 4,782 records, 785 were removed as duplicates and 3,959 were excluded following screening of titles and abstracts. Out of the 38 full text articles identified, 29 articles were excluded, and an additional four articles were identified from references or gray literature (Figure 2). A total of 13 articles met the inclusion/exclusion criteria, each describing a technique for evaluating quality of decision making and assessing a total sample of 2,400 subjects (individuals, organizations, or medicines).

### Part 2: identified techniques for evaluating quality of decision making

Out of the 13 techniques identified in this review, seven were developed specifically to assess decision making in the area of medicines' development, regulatory review, or HTA; two examined corporate decision making, and four were regarding general decision making. An examination of subject type demonstrated that the largest proportion of the techniques (6, 46%) assessed decision making of individuals, followed by the perception of individuals regarding the decision making of the organization (3, 23%) and then the decision making regarding the medicine itself (2, 15%). Only two techniques (15%) evaluated both the decision making of individuals and organizations.

Regarding the ability for each technique to evaluate the 10 QDMPs, the 13 techniques assessed a median of six QDMPs, with a mode of three QDMPs and only two techniques accounted for all 10 QDMPs. An examination of the two most commonly assessed practices indicated that 10 approaches assessed QDMP four (Evaluate both internal and external influences/biases), whereas nine approaches evaluated QDMP 1 (Have a systematic, structured approach to aid decision making). The two practices that were least evaluated were QDMP 9 (Ensure transparency and provide a record trail) and QDMP 10 (Effectively communicate the basis of the decision), with four and five approaches, respectively.

The 13 techniques are listed in Table 2 in descending order of total number of QDMPs evaluated by the technique, followed by the year of publication. They are then described in more detail based on published information.

**Table 2:** **Summary characteristics of the 13 techniques for assessing quality decision making listed in descending order of total QDMPs evaluated by the technique**.

**No**.	**Refernces**	**Title**	**Decision area**	**Study subject**	**Subject type**	**Method**	**QDMPs evaluated**									
							**1**	**2**	**3**	**4**	**5**	**6**	**7**	**8**	**9**	**10**
1	Matheson and Matheson, 1998	Organizational IQ test	Corporate	Industry (including pharm.)	Org.	45-item questionnaire assessing nine principles for strategic decision making in an organization (*N* = 100s)	✓	✓	✓	✓	✓	✓	✓	✓	✓	✓
2	Donelan et al., 2016	Quality of Decision Making Orientation Scheme (QoDoS) instrument	Medicines R&D/Reg. Review	Regulatory agency + pharm. industry	Org. + Ind.	Questionnaire with 47 items assessing organizational decision making culture and approach, as well as individual competence and style (*N* = 76)	✓	✓	✓	✓	✓	✓	✓	✓	✓	✓
3	Mindtools, 2013	“Good Are Your Decision-Making Skills?” Questionnaire	General	ND	Ind.	Questionnaire, “How Good Are Your Decision-Making Skills?” containing 18 items (*N* = ND)	✓	✓	✓	✓	✓	✓	✓	✓	✓	
4	Garbuio et al., 2015	Survey on strategic decision making	Corporate	Industry (including pharm.)	Org.	Survey (28 items) assessing relationship between robustness of analysis, dialogue, and decision-making effectiveness (*N* = 634)	✓	✓	✓	✓	✓	✓		✓		✓
5	Open University, 2013	Decision making Questionnaire	General	ND	Org. + Ind.	Questionnaire containing 12 items in three areas: decision-making process, psychological perspective and the role of social influences (*N* = ND)	✓		✓	✓	✓	✓	✓	✓		
6	Fischer et al., 2011	A structured tool to analyze coverage decision making	HTA	HTA agencies	Med.	Ten indicators for a structured empirical comparison of coverage decisions with corresponding ordinal rankings (*N* = 6)	✓	✓	✓					✓	✓	✓
7	Wood, 2012	Study exploring individual differences in decision-making styles as predictors of good decision making	General	University students	Ind.	Three part study: 1. General Decision-Making Style measure (25 items) 2. The BFI personality test (50 items) 3. Peer ratings of decision-making quality (26 items). (*N* = 315)	✓		✓	✓	✓	✓	✓			
8	Blenko et al., 2010	Decision and organizational scorecard	General	ND	Org.	Two web-based questionnaires, with 4 and 10 items respectively assessing decision effectiveness and organizational drivers (*N* = 1,065)	✓	✓								✓
9	Cowlrick et al., 2011	Questionnaire for assessing perception of risk through phases of medicine R&D	Medicines R&D	Pharm. industry	Ind.	Questionnaire with five sets of judgment statements to assess case studies for four medicines (*N* = 52)				✓		✓	✓			
10	McIntyre et al., 2012	Questionnaire for assessing how US FDA Advisory Committee Members prepare and what influences them	Regulatory Advisory Committee	Regulatory agency (US FDA)	Ind.	26-item questionnaire assessing US FDA committees' preparatory practices, influencers and preferences (*N* = 101)				✓	✓		✓			
11	Marangi et al., 2014	Survey of the Italian Medicines Agency (AIFA) 2013	Regulatory Advisory Committee	Regulatory agency (AIFA)	Ind.	Questionnaire, “Survey AIFA 2013” consisting of 17 questions, 4 regarding participant information and 13 assessing influences on AIFA committees (*N* = 72)				✓	✓		✓			
12	Beyer et al., 2015	A field study using the Domain Specific Risk Taking (DOSPERT) scale and the Big Five Jackson Inventory (BFI) scale	Reg. review	Regulatory agency (EU)	Ind.	Three part questionnaire: 1. Demographic data and DOSPERT scale; 2. Medicine case Study; 3. The BFI personality test consisting of 44 items to assess risk perceptions of assessors (*N* = 75)				✓		✓				
13	Salek et al., 2012	Scorecards to assess the quality of a regulatory submission and its review	Reg. submission and review	Reg. agencies + pharm. industry	Med.	Two scorecards containing 50 items grouped into seven domains: application format, content of the dossier, labeling, scientific advice, conduct of the review, communication, and overall assessment (*N* = 4)	✓									

*US FDA, Food and Drug Administration; HTA, health technology assessment; ind., individual; med., medicine; N, sample subject size used in testing; ND, not defined; org., organization; pharm., pharmaceutical; QDMP, quality decision-making practice; reg., regulatory; R&D, research and development; ✓, QDMP evaluated by the technique; the QDMPs are: 1, Have a systematic, structured approach to aid decision making (consistent, predictable and timely); 2, Assign clear roles and responsibilities (decision makers, advisors, contributors); 3, Assign values and relative importance to decision criteria; 4, Evaluate both internal and external influences/biases; 5, Examine alternative solutions; 6, Consider uncertainty; 7, Re-evaluate as new information becomes available; 8, Perform impact analysis of decision; 9, Ensure transparency and provide a record trail; 10, Effectively communicate the basis of the decision*.

#### Organizational IQ test (Matheson and Matheson, 1998)

This test measures an R&D organization's adherence to the nine principles of a smart organization: value creation culture, creative alternatives, continual learning, embracing uncertainty, outside-in strategic perspective, systems thinking, open information flow, alignment and empowerment, and disciplined decision making. The aim of this test is to benchmark “organizational intelligence” (i.e., the strategic decision-making abilities of an organization), identify barriers to decision quality, and prioritize the principles an organization must focus on to increase its performance (Matheson and Matheson, 1998).

The nine principles were developed based on previous studies of decision quality and best practices, which were conducted in five well-described phases, namely: brief survey to identify organizations that exemplified high quality R&D decision making; in depth interviews with 22 companies to identify 45 best practices; questionnaire to create statistical benchmarks for practices; validation through conformational studies with other companies; and extension of results to develop the principles. Subsequently, the test was developed and it consists of 45 questions, with five questions on each of the nine principles (Matheson and Matheson, 1998) These 45 questions can be used to evaluate all 10 QDMPs from the point of view of an organization.

The tool has now been used to assess hundreds of corporations from the point of view of thousands of individuals regarding their organization's decision making, and the results show that a strong IQ profile correlates positively with financial performance of organizations, thereby demonstrating the applicability of the tool (Matheson and Matheson, 2011). The sensitivity or reproducibility of the tool has not been described. Although the tool evaluates the full spectrum of organizational-level QDMPs, the practices of an individual are not evaluated. Furthermore, the test is specific to R&D organizations and possesses generalizability to be applied within different departments in companies including the pharmaceutical industry, but its design did not involve input from regulatory authorities or HTA agencies, and consequently, it does not assess the issues specific to those stakeholders.

In summary, the Organizational IQ, which was developed through studies with a range of R&D organizations, is a 45-item test that measures corporate decision making across all 10 QDMPs from an organizational point of view only; it has been assessed in >100 companies, but it has not been developed or validated within agencies.

#### Quality of decision making orientation scheme (QoDoS; Donelan et al., 2016)

QoDoS is a generic instrument for assessing the quality of decision making. Its aim is to evaluate the quality of decision making of individuals and organizations in order to promote awareness of best practices and biases in decision making. This should facilitate a clearer understanding of strengths and areas for improvement, and ultimately encourage a better decision-making process for both companies and agencies (Donelan et al., 2016).

The tool was developed and validated using a standardized approach with both qualitative and quantitative techniques. The qualitative phase involved semi-structured interviews with 29 key opinion leaders from the pharmaceutical industry (10), regulatory authorities (9), and contract research organizations (10) (Donelan et al., 2015). This was followed by content validity testing, using a panel of experts for language clarity, completeness, relevance, and scaling, resulting in a favorable agreement by panel members with an intra-class correlation coefficient value of 0.89 (95% confidence interval = 0.56, 0.99). The quantitative phase of factor analysis produced a 47-item tool with four domains: Part I = Organizational—Decision-Making Approach and Decision-Making Culture; Part II = Individual—Decision-Making Competence and Decision-Making Style. QoDoS showed high internal consistency (*n* = 120, Cronbach's alpha = 0.89), high reproducibility (*n* = 20, intra-class correlation = 0.77) and a mean completion time of 10 min (Donelan et al., 2016). Most importantly, QoDoS items can be mapped to assess the full spectrum of the 10 QDMPs across different decision points from both the perspective of an organization and an individual.

The applicability of the tool in a regulatory authority and pharmaceutical company setting was confirmed through a study with 76 participants (50% from regulatory authorities and 50% from pharmaceutical companies). The findings of this pilot study demonstrate that QoDoS has the practicality to identify differences in decision making between individuals and their organization as well as between companies and regulatory authorities across all 10 QDMPs. Moreover, QoDoS possesses strong psychometric properties, is easy to understand, and can be completed in a short time frame. Nevertheless, the tool needs to be further tested in terms of its sensitivity and reliability, as well as validated in HTA agencies regarding decision-making practices during the reimbursement of medicines as well as evidence submission to support reimbursement of medicines in pharmaceutical company departments (Bujar et al., 2016b).

To summarize, the QoDoS is a 47-item test that measures quality decision making across all 10 QDMPs from an organizational and individual point of view. It was developed through studies with the population it was intended for (i.e., both the pharmaceutical industry and regulatory authorities); its applicability has been assessed with 76 participants with more testing planned in the future.

#### “How good are your decision-making skills?” questionnaire Mindtools, 2013

The aim of this generic web-based questionnaire was to assess individual decision-making skills and practices. It is composed of 18 questions that relate to six essential steps in any decision-making process: establishing a positive decision-making environment, generating potential solutions, evaluating the solutions, deciding, checking the decision and communicating and implementing (Mindtools, 2013).

The method used in the development or validation was not published, nor any results that were collected from participants. Overall, this questionnaire assesses nine QDMPs from the point of view of an individual, but nevertheless does not assess QDMP 10 regarding communication of the decision, and it does not assess the personal perceptions regarding organizational decision making. This tool possesses generalizability to other decision areas and subjects, but it lacks published data on its method used in development or validity testing to determine its practicality and robustness.

In summary, this questionnaire is a generic 18-item test that measures decision making across nine QDMPs from an individual point of view only; its origin and testing were not described.

#### Survey on strategic decision making (Garbuio et al., 2015)

The aim of this survey was to assess strategic decision making amongst international companies, including the pharmaceutical industry. It was used in a study to test three hypotheses regarding the effect of two dimensions, namely the analysis performed on the decision and strategic conversations about the decision (coined “disinterested dialogue”) on decision-making effectiveness (Garbuio et al., 2015).

The development of the survey was based on a literature review, previous scholarly works in this area (Dean and Sharfman, 1996), and interviews with 29 executives from large corporations. The survey assesses an individual's perception of decision making and contains a total of 28 questions focusing on a key strategic decision made in the past 5 years: eight on demographic characteristics of the respondents, six variables measuring robustness of analysis performed, six on disinterested dialogue, four on strategic decision effectiveness and four control variables. The Cronbach's alpha coefficient for strategic decision effectiveness, robustness of analysis, and disinterested dialogue were 0.886, 0.793, and 0.716, respectively, which indicates the survey was appropriately formulated and suitable for the analysis (Garbuio et al., 2015). These survey items can be mapped to 8 out of the 10 QDMPs from the point of view of an individual, mainly regarding “Structure and Approach” (QDMP 1, 2), “Evaluation” (QDMPs 3, 4, 5, and 6), “Impact” (QDMP 8) as well as communication of the decision (QDMP 10). It nevertheless does not assess QDMP 7 regarding re-evaluating the decision with new information, as well as QDMPs 9 regarding ensuring transparency and providing a record trail.

The survey was sent to 5,210 executives from a global range of industries, regions, and functional specialties. The response rate was 45%, which may be due to the lengthy method used in the study. Exploratory and confirmatory factor analysis was used to test the hypotheses (Garbuio et al., 2015). Overall, this survey provided a good overview of some of the perceptions of organizational-level QDMPs in companies. Nevertheless, the survey does not evaluate two of the QDMPs, and it does not assess the individual level decision-making practices, which could have provided further key insights regarding decision making in companies. The survey possesses generalizability to be used in various teams and departments in pharmaceutical companies, but would need to be validated further in terms of its sensitivity and reproducibility. Nevertheless it is not appropriate for use in regulatory authorities and HTA agencies due to the specific nature of the technique.

To summarize, this technique is a 28-item survey that assesses corporate strategic decision making from the point of view of an organization. Consequently it was developed through studies with just companies (some of which were pharmaceutical) and subsequently tested with 634 subjects across eight QDMPs; it is not relevant to agencies.

#### Open university decision-making questionnaire (Open University, 2013)

This test was administered as part of a postgraduate course in Business at the Open University, UK. The aim of the questionnaire was to help an individual develop greater insight into their personal decision-making processes (Open University, 2013).

The development or testing of the tool was not published. The questionnaire is composed of 12 questions assessing decision making in a recent major decision concentrating on three areas: formal rational decision-making process; psychological perspective and focus on the tendency to rely on “heuristics” (i.e., subjective judgments); and the role of social influences on decision making. The 12 questions can be mapped to 7 out of the 10 QDMPs from the point of view of an individual (though a small number of questions are applicable to organizations too), excluding QDMP 2 regarding roles and responsibilities and QDMPs 9 and 10 regarding transparency and communication.

In summary, this technique is a 12-item questionnaire that assesses decision making from the point of view of an individual or an organization. Due to its generic nature, it may be applicable to companies and agencies alike, though this would require practicality testing and validation. Nevertheless, the development and testing of the tool were not described and the questionnaire assessed 7 out of 10 QDMPs.

#### A structured tool to analyze coverage decisions (Fischer et al., 2011)

This study presents a structured tool that aims to analyze coverage decision-making processes and drivers. Its purpose was to compare country-specific reimbursement systems to inform a number of stakeholder including HTA agencies, manufacturers, policy-makers, patients, and the public (Fischer et al., 2011).

The tool was developed based on the published conceptual framework of Rogowski et al. (2008) that identified seven key components in deciding on the reimbursement of a new technology. Fifteen semi-structured interviews were conducted to apply this framework to specific case studies in the area of cancer prevention with participants from decision-making institutions from Austria, Sweden, and Lithuania; data were further validated with publicly available documentation. From the case studies, the structured scheme describing the components of reimbursement decision processes and a proposal of ordinal rankings were deduced and validated through consultations with experts. The developed scheme contains eight reimbursement decision-making steps namely: trigger; participation, publication, assessment, appraisal, reimbursement, management, and impact (Fischer et al., 2011). Overall, the tool evaluates six of the QDMPs covering all practices relating to “Structure and approach,” “Impact,” and “Transparency and Communication,” but only one QDMP regarding “Evaluation,” namely QDMP 3 (assign values and relative importance to decision criteria).

This method was applied to case scenarios with six medicines and it generated a scheme for structured and consistent comparison of a large variety of procedural aspects of reimbursement decision processes. The study met its purpose and a robust method was used to develop the scheme. Nevertheless, the semi-structured phone interviews were considered time consuming and the scope for interpretation of questions during interviews was wide. Further validation of the structured scheme and indicators as well as development of a web-based tool for more efficient large-scale empirical studies is still needed (Fischer et al., 2011). Moreover, the scheme does not explicitly assess QDMPs relating to evaluation, which if incorporated, would perhaps give more rationale for some of the heterogeneity seen in the decision outcomes.

In summary, this technique, which was designed based on a conceptual framework, is a structured tool to analyze coverage decision making of medicines using 10 indicators across 6 QDMPs. Nevertheless, the tool is specific to evaluating certain decisions regarding technologies in HTA agencies, and was not designed to assess general organizational or individual practices or decision making within companies and regulatory authorities.

#### Study exploring individual differences in decision-making styles as predictors of good decision making (Wood, 2012)

The aim of this study was to examine the relationship between decision-making styles and subjective self- and peer-ratings of decision quality. The second purpose of the study was to evaluate the incremental validity of decision styles and personality traits for predicting decision quality (Wood, 2012).

The method involved three phases. Decision style was measured using Scott and Bruce (1995) General Decision-Making Style measure; the Big Five Jackson Inventory personality test (50 items) was conducted using the International Personality Item Pool short scales (Goldberg, 1999; Goldberg et al., 2006) and peer ratings of decision-making quality were collected using a scale where peers were asked to evaluate their friends' decision making by rating their habits in four sections. The first two measures were developed and validated previously; the third was created for the purpose of this study through factor analysis and psychometric tests for internal consistency. Overall, this technique can be used to assess 6 out of 10 QDMPs, including all 5 QDMPs relating to evaluation, as well as QDMP 1 regarding having a structured approach. The technique can be used to assess the decision making of individuals only, but uniquely, the assessment can be conducted from the point of view of the participants as well as the perception of peers regarding the ratees' decision-making habits.

Three hundred and fifteen target participants from undergraduate courses at a public university in the Midwestern United States took part in phases 1 and 2 of the study, using an online survey administration and data collection system. In addition, 168 peer raters completed phase 3 of the study regarding decision-making habits of the target participants. However, the completion time was not specified, and there are limitations to the use of the peer rating system, as indicated by relatively low response rate (53% of phase 1 and 2 participants participated in phase 3). Furthermore, this technique does not assess organizational-level QDMPs or individual practices regarding roles and responsibilities, decision impact, transparency, and communication, as specified by QDMPs 2, 8, 9, and 10, respectively.

To summarize, this three part study, which aimed to assess differences in decision-making styles, was designed to be global in nature, and consequently it may be applicable to any decision areas as well as be used by any subjects. Nevertheless, it assessed only 6 QDMPs and the method would require testing and validation in pharmaceutical companies, regulatory authorities, and HTA agencies.

#### Decision effectiveness and organizational scorecards (Blenko et al., 2010)

The aim of the scorecards was to give a high level assessment of decision making within organizations, help identify the most pertinent issues, and guide prioritization of actions on specific decisions and broader organization enablers. The 2 scorecards represent step 1, namely “Score your organization,” with a sequence of five steps to improve decision making (Blenko et al., 2010).

The method used in the development of the 2 scorecards was not published. The decision effectiveness scorecard is composed of four items assessing quality, speed, yield, and effort of the decision making-process. The organizational scorecard has 10 items and it is used to assess drivers of decision effectiveness in an organization, namely context, alignment, accountability, structure, process, information, tools, skills and capabilities, leadership, and culture. Overall, the 2 scorecards look beyond decision making as a marker of an effective organization, and consequently assess 3 out of 10 organizational-level QDMPs, namely QDMP 1 (structure), 2 (roles and responsibilities), and 10 (communication of the decision).

The scorecards were used to assess 1,065 organizations including large multinational corporations, entrepreneurial ventures, research universities, and non-profit institutions, thereby highlighting the generalizability of this method (Blenko et al., 2010).

To summarize, this two-part scorecard is generic in nature and can be used to assess decision making of organizations. Nevertheless, the approach used in the development and validation of the scorecards was not described, and none of the individual-level QDMPs and only three organizational-level practices can be assessed.

#### Questionnaire for assessing perception of risk through phases of medicine R&D (Cowlrick et al., 2011)

This questionnaire was designed to assess risk perceptions in the pharmaceutical industry and allied healthcare sectors, and is analogous to the Beyer regulatory authority study (Beyer et al., 2015) described later in this manuscript. The aim is to investigate go/no-go judgments in discovery and medicine development in order to evaluate the influence of personality, experience as well as demographic traits on decision making (Cowlrick et al., 2011).

The method consists of a web-based questionnaire where respondents were asked to make five sets of judgment within case studies regarding four medicines derived from real scenarios. These five judgments were derived from 18 non-discrete steps relating to the regulatory requirements as set by the European Medicines Agency (EMA) and the US Food and Drug Administration (US FDA) and were related to major key segments in medicines R&D, namely target selection/ pharmacology; toxicology; biopharmacy and galenics; and clinical development/market introduction. In addition, the study assessed to what extent the individual judgments given by the respondents were influenced by demographics, experience, or their perceived entrepreneurial character. Paradigms of entrepreneurial behavior were selected based on previous research, although the exact process for question design was not described (Cowlrick et al., 2011). The completion of the questionnaire takes ~10 min. Overall, the tool assesses only 3 of the 10 QDMPs relating to evaluation, namely QDMP 4 (evaluate internal/external influences/biases), 6 (consider uncertainty), and 7 (re-evaluate as new information becomes available). Due to the stepwise nature of the cases, this tool offers the potential to understand whether individuals re-evaluated their decision making with new information, which was not possible in the single step decision study by Beyer et al. (2015).

The questionnaire was completed by 52 participants, a response rate of 62%, which indicated moderate acceptability (Cowlrick et al., 2011). The authors did not describe how the method used was developed, compared with the Beyer et al. (2015) study in which validated tools were used, but nevertheless, this technique was less time intensive.

In summary, this questionnaire was used to assess decision making during the medicines R&D across three QDMPs from the point of view of an individual only. The design of the questionnaire is unknown. Furthermore, the questions are generalizable to other stakeholders, whereas the case studies are specific to the pharmaceutical industry and would require major modifications and further validation if to be used in regulatory authorities and HTA agencies.

#### Questionnaire for assessing how US FDA advisory committee members prepare and what influences them (McIntyre et al., 2012)

This qualitative study was carried out to understand preparatory practices, influencers, and preferences of US FDA advisory committees regarding materials provided by the sponsor and the FDA, advisory committee presentations and Q&A sessions. The goal was to understand what advisory committee members want from sponsors to enable their informed participation in the meetings (McIntyre et al., 2012).

It consisted of a web-based survey composed of 26 questions, with a target completion time of 10 min. The method used in the design and validation of the questions was not described. This survey assesses a limited set of decision-making practices, namely QDMP 4, 5, and 7 regarding evaluation of influences/biases, examination of alternatives and re-evaluation with new information respectively from the point of view of an individual only.

The qualitative questionnaire was administered to 101 current or former members of one of the US FDA public biomedical advisory committees. The advantages are a short completion time and that the questionnaire captured the relevant individual practices and influences regarding evaluation of material for committee meetings (McIntyre et al., 2012). Moreover, the survey does not evaluate 7 out of the 10 individual practices, or what the individuals think about the practices of the organization (in this case, the committee). This is likely due to the fact that this was outside the scope of this study.

In summary, this 26-item questionnaire was used to assess decision making of the US FDA Advisory Committee across three QDMPs from the point of view of an individual only. Nevertheless, the development and validation of the questions were not published. Although this questionnaire could be adopted for other regulatory bodies, the questions are specific to committee decision making and have limited transferability to other regulatory areas as well-stakeholders such as industry and HTA agencies.

#### Survey of the italian medicines agency (AIFA) 2013 (Marangi et al., 2014)

A questionnaire that was analogous to US FDA's was undertaken by AIFA in 2013 to assess the influences on agency committees and secretariats' opinions and decisions. This study was part of an initiative to enhance a transparency-oriented policy and improve information exchange as well as decision making with stakeholders (Marangi et al., 2014).

The study was a web-based questionnaire with a completion time of 7 min and a total of 17 questions assessing the demographics, professional qualifications, and committee experiences, followed by questions regarding internal and external influences on committees and secretariat member opinions. The method used in the design of the questions was not described. Similar to the US FDA study, the questions are specific to AIFA advisory committee meetings and the survey assesses a narrow set of individual QDMPs relating to decision-making evaluation practices only (QDMP 4, 5, and 7).

A total of 72 participants from AIFA committees, secretariats, and subcommission members took part in the study (Marangi et al., 2014). This qualitative survey can be completed in a short timeframe, but the development of the questions was not described.

To summarize, this 17-item questionnaire was used to assess decision making of the AIFA Advisory Committee across three QDMPs from the point of view of an individual only. Moreover, the origin of the questionnaire has not been published. Although the questionnaire meets its objectives, it is not transferable outside the regulatory advisory committee setting to industry and HTA agencies.

#### A field study using the domain-specific risk taking (DOSPERT) scale and the big five jackson inventory (BFI) scale (Beyer et al., 2015)

This set of tests aimed to assess the influence of risk attitudes and personality traits on clinical decision making of expert regulators. The two main objectives of the study were to describe the distribution of risk attitudes among medical assessors within EMA and to measure their personality traits and cross-domain risk attitudes (Beyer et al., 2015).

This study was implemented as a web-based questionnaire and was composed of three well-defined phases using validated tests; phase 1: demographic data and 30-item DOSPERT scale to measure risk appetite; phase 2: medicine case study using mock “clinical dossiers” for three medicines and eight rating scales on benefit-risk dimensions; and phase 3: The BFI 44-item personality test. Ordinal regression models were used to evaluate the relationships between the variables regarding risk taking, personality as well as the assessment of benefit and risk of a medicine (Beyer et al., 2015). Although the study evaluates the relationship between perception of uncertainty and personal influences as defined by QDMP 4 and 6, it does not evaluate any of the practices relating to decision making “structure and approach,” “impact,” or “transparency and communication” of decision making. Indeed other individual as well as organizational decision-making practices should be explored in order to understand the broader context of these findings.

This technique was used to assess 75 assessors from European regulatory authorities. It utilizes validated methods and it meets its purpose of assessing risk attitudes in medical assessors (Beyer et al., 2015). It also possesses generalizability to be tested in other agencies, but would need to be adapted for companies and HTA agencies with some modifications to phase 2 (medicine case study). A major drawback of this technique is that it is resource and time intensive for both the assessors and researchers.

To summarize, this three part questionnaire was used to assess decision making of regulatory assessors. The development of the study was well-described, but it can only be used to assess 2 out of 10 QDMPs and it does not evaluate the perceptions of the individuals regarding their organization.

#### Scorecards to assess the quality of a regulatory submission and its review (Salek et al., 2012)

Two scorecards were developed to enable companies to assess the quality of the regulatory review and for regulatory authorities to assess the quality of submission relating to a specific product. This allows for a unique comparison of the quality of the submission compared to review, as well as inter-product, company, or agency cross-evaluations (Salek et al., 2012).

The scorecards were developed through a structured and well-described process of conceptualization (expert discussions; bibliography review), item generation (literature review; expert input), and reduction (qualitative reduction; content validation). Each scorecard includes more than 50 items that are grouped into seven domains (application format, content, labeling, scientific advice, conduct of the review, communication, and overall assessment). The 2 scorecards enable a quantitative assessment of the quality of the information and communication specific to dossiers and go beyond just decision making as a marker of quality (Salek et al., 2012). As a consequence they evaluate whether a structured approach was taken (QDMP 1), but do not evaluate the roles and responsibilities of an individual/organization (QDMP 2) or the quality of individual and organizational decision making as outlined in QDMP 3, 4, 5, 6, and 7, which are related to the evaluation practices as well as preferences, or QDMP 8, 9, and 10 which assess decision-making transparency and decision communication.

The scorecards were tested by three major regulatory authorities and four international pharmaceutical companies based on the same four products. The majority of respondents agreed that the scorecards covered all critical factors that affect the quality of the dossier and the review. A number of modifications were made following a pilot study, particularly the inclusion of definitions for each rating response option, which further added to the robustness of the scorecards (Salek et al., 2012).

The high response rate as well as positive feedback indicated their practicality, clarity, and applicability, whilst the method used was well-described. The scorecards can be used for different regulatory procedures and across different teams, and can be therefore applied to optimize the regulatory authority and company processes. Importantly, this technique encourages open internal and external dialogue. Although the scorecards meet their purpose of assessing the quality of review and submission, it can be argued that aspects specific to decision making, other than having a structured approach (QDMP 1), need to also be addressed to ensure that companies and agencies are not only embedding good review and submission practices, but are also making quality decisions (Liberti et al., 2013).

In summary, the scorecards can be used to assess the quality of regulatory submission and review, and were developed using a well-defined framework using input from regulatory authorities and companies. Nevertheless, they only assess one QDMP and are outside the scope of HTA agencies; a separate set of scorecards would need to be developed and validated for this purpose.

### Part 3: measurement properties of the techniques for evaluating quality of decision making

The 13 techniques were evaluated in terms of their measurement properties, according to six key criteria (McDowell, 2006; Streiner et al., 2015), namely theoretical underpinning (development technique was based on a well-described methodological framework); psychometric properties (development of technique involved psychometric tests, content validity, and internal consistency); psychometric evaluations (the validity, reliability, and sensitivity of the tool was demonstrated); demonstrated practicality (the technique was applied to target population through pilot studies); generalizability (the technique can be used across industry, regulatory, and HTA); and applicability (the technique is applicable to evaluating individuals and organizations). The techniques were listed in a descending order by total number of criteria met, followed by year of publication (Table 3). Only five properties are shown in Table 3, as none of the techniques underwent psychometric evaluations.

**Table 3:** **Measurement properties of the 13 techniques for assessing quality decision making in descending order of total properties met, followed by year of publication**.

**No**.	**References**	**Title**	**Theoretical underpinning**	**Psychometric properties**	**Demonstrated practicality**	**Generalizability**	**Applicability**
			**Development technique was based on a well-described methodological framework**	**Development of technique involved psychometric tests (content validity and internal consistency)**	**The technique was applied to target population through pilot studies**	**The technique can be used across industry, regulatory, and HTA**	**The technique is applicable to evaluating individuals and organizations**
1	Donelan et al., 2016	Quality of Decision Making Orientation Scheme (QoDoS) instrument	✓	✓	✓	✓	✓
2	Wood, 2012	Study exploring individual differences in decision-making styles as predictors of good decision making	✓	✓	✓	✓	
3	Garbuio et al., 2015	Survey on strategic decision making	✓	✓	✓		
4	Matheson and Matheson, 1998	Organizational IQ test	✓		✓		
5	Blenko et al., 2010	Decision and Organizational Scorecard			✓	✓	
6	Fischer et al., 2011	A structured tool to analyze coverage decision making	✓		✓		
7	Cowlrick et al., 2011	Questionnaire for assessing perception of risk through phases of medicine R&D	✓		✓		
8	Salek et al., 2012	Scorecards to assess the quality of a regulatory submission and its review	✓		✓		
9	Open University, 2013	Decision making Questionnaire				✓	✓
10	Beyer et al., 2015	A field study using the Domain Specific Risk Taking (DOSPERT) scale and the Big Five Jackson Inventory (BFI) scale	✓		✓		
11	McIntyre et al., 2012	Questionnaire for assessing how US FDA Advisory Committee Members Prepare and What Influences Them			✓		
12	Mindtools, 2013	“How Good Are Your Decision-Making Skills?” Questionnaire				✓	
13	Marangi et al., 2014	Survey of the Italian Medicines Agency (AIFA) 2013			✓		

Out of the 13 articles, only one met all criteria described in Table 3. Eleven (85%) of the techniques met at least two criteria, namely demonstrated practicality, followed by theoretical underpinning (8, 62%). On the other hand, the criteria that were met by the minority of the techniques were generalizability of study subjects (5, 38%); psychometric properties (3, 23%); and applicability to both assessing individuals and organizations (2, 15%). None of the 13 techniques met the criteria of psychometric evaluations regarding the demonstration of sensitivity/responsiveness (detecting change over time), construct validity (demonstrating strong correlation with closely related measures—i.e., convergent validity; and poor correlation with distantly related measures—i.e., divergent validity); and reliability (producing similar results under consistent conditions) and were consequently not illustrated in Table 3.

## Discussion

This is the first literature review that has identified techniques for evaluating the quality of the decision-making process in medicines' development, regulatory review, and HTA. The objectives were to compare the existing techniques, assess their properties, identify research gaps, and recommend the next steps.

This literature review has demonstrated that the area of quality decision making has been explored to a certain extent, but in a fragmented way, where studies have been independent, few have been replicated, and there is no overarching mutually agreed conceptual framework.

This is consistent with previous research which has identified that the majority of pharmaceutical companies and regulatory authorities do not have formal assessments in place to periodically measure the quality of their decision making (Bujar et al., 2016a) and this could be partially explained by the fact that very few appropriate techniques exist to enable this to be done. Nevertheless, both of these stakeholders believe that such measurements of quality decision making would be possible and would improve practices for individuals and organizations, which could be achieved by utilizing the best techniques currently available (Bujar et al., 2016a). Consequently, there is a need to identify a technique that is relevant, robust and can be applied to pharmaceutical companies, regulatory authorities and HTA agencies.

It should be noted that most of the techniques are suitably robust to be used in the area they were created for, and therefore should be utilized for their specific purposes despite not having the applicability across all three stakeholders. Nevertheless, an advantage of having a tool valid across all three stakeholders would be the ability to discuss, share and compare challenges in decision making using a common terminology. Already companies, regulatory and HTA agencies have been collaborating regarding topics such as parallel scientific advice (EMA, 2016), real world evidence generation (McAuslane et al., 2016a) as well as parallel regulatory and HTA reviews (McAuslane et al., 2016b), and it would be of interest to align best practices in decision making across the three groups.

### Key trends and features of existing techniques

This review identified 13 techniques for evaluating the quality of decision-making process in medicines' development, regulatory review, and HTA. This is a relatively low number, considering the increasing pressure on pharmaceutical companies, regulatory authorities, and HTA agencies to make the best-quality decisions (Liberti et al., 2013). Moreover, routine assessment of the quality of the decision-making process (as opposed to just measuring outcomes) has been recognized as key for improving the productivity of any organization (Kahneman, 2011).

Out of the 13 techniques, 1, the Organizational IQ, was developed in 1998, and 12 were published from 2010 onward. This indicates that although some initial work was done early on and stands ahead of its time, it is only in more recent years that measuring decision making or understanding decision making styles and approaches has become of interest in this arena.

The 13 techniques have unique aims as well as strengths and weaknesses based on their origin and the methods that were used in their development and testing. Furthermore, they can be classified into three groups described below.

#### Group 1: seven specific research techniques for assessing the quality of the decision making-process in medicines' development, regulatory review, or HTA

An examination of the seven techniques developed specifically to assess decision making in the area of medicines' development, regulatory review, or HTA demonstrated that a number of were developed to meet the needs of a particular organization, for example to increase transparency of regulatory advisory committee meetings within AIFA and US FDA (McIntyre et al., 2012; Marangi et al., 2014). Nevertheless, although these two surveys are practical for their purpose and are characterized by low resource intensity, their design was not described and their latitude for generalizability and measurement against the QDMPs is limited due to their specific scope.

Other techniques, as exemplified by the work of Beyer et al. (2015) and Cowlrick et al. (2011) were developed as research tools to measure the effect of risk attitudes and personality traits on decision making regarding medicines' development and review. These studies developed and tested a number of interesting hypotheses, but are resource intensive, and are limited to the specific decision area as well as to the subjects they were designed to assess. Finally, a number of techniques evaluate more than just decision making as a marker of quality, such as the “Scorecards to Assess the Quality of Regulatory Submission and Review” (Salek et al., 2012) and the “Structural tool to analyze coverage decision making” (Fischer et al., 2011) and may represent promising techniques to study the general area of quality to assess the effectiveness of an organization or an outcome (Fischer et al., 2011; Salek et al., 2012).

The last technique in this group “Survey on strategic decision making” (Garbuio et al., 2015) looked specifically at corporate decision making, which is applicable to pharmaceutical companies (i.e., medicines' development). Although the tool was developed based on a well-designed methodological framework and demonstrated practicality in companies, it is resource intensive and does not possess the generalizability to allow its application to other subjects such as regulatory authorities or HTA agencies or to individuals (as opposed to just organizations) due to the specific nature of the questions. It is important to note that, all seven of these techniques were used primarily for research or as one-off studies, but they have not been systematically adopted for use by companies, regulatory authorities or HTA agencies. Overall, none of these seven techniques are appropriate to measure decision making quality due to low generalizability as well as an inability to measure all 10 QDMPs.

#### Group 2: four educational or consulting techniques for assessing the quality of the decision-making process

Four of the techniques were either developed for educational purposes or by consulting groups to assess decision making in general (Blenko et al., 2010; Wood, 2012; Mindtools, 2013; Open University, 2013). Consequently, although all four possess good generalizability to be applied to industry, regulatory authorities, and HTA agencies, only one of the techniques has published information regarding its design and psychometric properties (Wood, 2012), while only two have demonstrated practicality through pilot studies (Blenko et al., 2010; Wood, 2012). As well as that, only one of the techniques has the applicability to measure decision making in both individuals and organizations (Open University, 2013). Consequently, these may be useful generic tools for informal assessments of decision making, but due to lack of published data regarding their design and measurement properties, as well as a lack of applicability to individuals and organizations across the 10 QDMPS, these techniques lack robustness to formally evaluate quality decision making in companies, regulatory authorities, and HTA agencies.

#### Group 3: the two most promising techniques for assessing the quality of the decision-making process

Only 2 of the 13 techniques evaluated the full spectrum of the 10 QDMPs, namely the Organizational IQ (Matheson and Matheson, 1998) and QoDoS (please see the Appendix in Supplementary Material; Donelan et al., 2016). Incidentally, the two instruments possess a similar number of items (45 for Organizational IQ and 47 for QoDoS) and can therefore be completed in a short timeframe. Both techniques were designed based on a well-hypothesized conceptual and well-described methodological framework and demonstrated practicality in the target populations, but only QoDoS underwent psychometric testing during its design, namely content validity and internal consistency. It is nevertheless interesting and significant that these two most promising techniques for quality decision making were developed independently, with a 20-year time gap between them and both resulted in similar key features.

The second area of disparity is that the Organizational IQ test, unlike QoDoS, does not assess the practices of both individuals and organizations, but just the latter. Although it could be argued that this is sufficient as individuals make up an organization, assessing individuals is also key as people tend to score themselves more favorably but be more critical of an organization (Bujar et al., 2016b). While this could be a potential sign of bias, areas of disparity between the individuals and organizations could also indicate deficiencies in practices within companies, agencies and committees, as changes in individuals could translate into better organizational practices. Consequently, assessment with QoDoS gives a unique perspective of both groups which helps to identify areas for improvement.

In the third area of divergence between the two techniques, the development of the Organizational IQ test was based on research among R&D organizations, and consequently the factors in decision making specific to regulatory authorities and HTA agencies were not incorporated into the instrument. Nevertheless the Organizational IQ test represents a practical approach and possibly a gold standard for measuring decision making of pharmaceutical and other companies. In contrast, QoDoS was developed specifically to look at decision making in the area of medicines' development and regulatory review, based on interviews with key opinion leaders from agencies and companies. It is therefore a more appropriate tool compared with the Organizational IQ test to measure quality decision making in pharmaceutical companies, regulatory authorities, and HTA agencies and moreover it can be applied to identify commonalities and differences between the various stakeholders as well as strengths and areas for improvement (Bujar et al., 2016b). Most importantly, it can increase an awareness of the biases and influences that need to be considered when making decisions, as well as the best practices that should be incorporated into a decision-making framework. Although the psychometric evaluation of QoDoS has been partially established, further testing is still required to demonstrate practicality in HTA agencies, as well as its construct validity, sensitivity and reliability.

### Emerging research themes

A secondary outcome of this review has been the identification of research themes and hypotheses regarding decision making preferences and influences. These have been derived from the pilot studies conducted using the identified techniques and instruments. A selection of the key findings is described below.

The research themes identified through the work of Beyer et al. (2015) as well as Cowlrick et al. (2011) relate to the impact of personality traits, functional role, education, and gender on decision making of individuals within pharmaceutical companies and regulatory authorities. Both studies demonstrate that these factors can explain the variability in judgments and decision making techniques within organizations. Moreover, the study by Beyer et al. (2015) demonstrated that “conscientiousness” (being thorough and careful) predicted an increase in the perception of a medicine's benefits, whereas extraverted disposition was predictive of seeing fewer risks, and interestingly, male assessors gave higher scores for a medicine's benefit ratings than did female assessors. Importantly, these research findings are in line with general research on decision making and risk taking (Thaler and Sustein, 2009; Lovallo and Sibony, 2010; Kahneman, 2011; SDG, 2011) and emphasize that despite sound scientific knowledge and experience, individuals within agencies and companies are equally prone to biases and reliance on emotional judgments when compared with lay people. Moreover, individuals are likely to rate their performance as superior to their organization (Bujar et al., 2016b). This has already been emphasized in a number of recent studies, which have discussed the role of informal factors in decision making, relating to biases and behaviors, which influence the decision-making processes during the delivery of dossiers for regulatory submissions as well as during the medicine evaluation process (Tafuri, 2013; Cook et al., 2014; Donelan et al., 2015). Furthermore, the occurrence of biases within organizations or their influence on decision making was perceived by regulatory authorities and pharmaceutical companies as one of the major barriers to ensuring quality decision making. This emphasizes the importance of implementing a decision-making framework and incorporating the 10 QDMPs, particularly making decision values, preferences and uncertainty more explicit, as suggested by pharmaceutical companies and regulatory agencies in order to ensure that quality decisions are made throughout the life cycle of medicines (Bujar et al., 2016a).

The two surveys described by McIntyre et al. (2012) and Marangi et al. (2014), which studied the decision making of the US FDA and the Italian AIFA Advisory Committees regarding how the members prepare for meetings and what influences their decision-making, interestingly both concluded a number of similar findings. Those included a diverse range of practices utilized by the members as well as potential biases that influence the decision making that may subsequently need closer monitoring. Both studies identified that a large proportion of individuals attend committee meetings having already decided how to vote. As well as that, the members are seldom influenced by external stakeholders such as healthcare professionals, patients, and sponsors, despite finding their input important. On the other hand, the members are frequently influenced by internal committee members, particularly colleagues or the committee chair. Both studies suggest that in addition to already minimizing biases due to conflicts of interest, the agencies should consider measuring the impact of the so-called intellectual bias on decision making during meetings, which may lead the committee members believing information which appears more favorable or familiar. Moreover, better practices could be achieved by implementing the 10 QDMPs into agency processes that promote having a structured approach to decision making (QDMP 1), assigning values to decision criteria (QDMP 3), evaluating different alternatives (QDMP 5), and new information (QDMP 7), and more importantly, evaluating different influences and biases (QDMP 4) to ensure that structured decisions are made during the review of medicines. It would be of interest to widen the scope of such studies to other regulatory agencies, as well as HTA committees in order to address the uncertainty surrounding the process for appraising whether or not medicines should be recommended for reimbursement (Calnan et al., 2017).

Finally, the research by Garbuio et al. (2015) assessed decision making amongst international companies through a study of 634 outcomes made by executives across multiple industries, including the pharmaceutical industry. This study demonstrated first of all that strategic decision making is important for decision effectiveness. Secondly, the study found that robust analysis of data and strategic conversations and communication during decision making around the data (“disinterested dialogue”) have a significant positive relationship with decision-making effectiveness. Moreover, the findings demonstrated that the strategic conversations have in fact more impact on decision effectiveness than analysis of data. This is consistent with previous research in this area, such as that by Westley (1990) where managers were interviewed regarding challenges on strategic making; for example the difficulties in not being included in strategic meetings but being given lengthy reports instead, as expressed by one of the interviewees: “just looking at the numbers doesn't give me the insights. It does not give me to total picture. I don't know how they (executives) are interpreting those numbers.”

This emphasizes the importance of QDMP 10 regarding communication during decision making, as well as QDMP 9 to ensure transparency and provide a record trail of the process through which the decision was made. Despite its apparent importance, QDMPs 9 and 10 were the least assessed practices by the 13 decision-making techniques identified in this review. Consequently these practices may require closer evaluation and better incorporation into the decision-making practices of individuals and organizations to ensure decision effectiveness.

### Research limitations

The present study was limited to existing, published techniques for assessing the quality of decision making using a web-based literature review of peer-reviewed journal articles found through sources described earlier. There may be other techniques or instruments that have not been formalized or published in the English language and were therefore not included. In addition, there may be data regarding the development and testing of the included techniques that have not been published, and which could have subsequently influenced the assessment of the tools.

## Conclusions

This review identified a general paucity of research in the area of decision making in medicines' development and review, but particularly in the area of HTA. This applies to the development as well as the systematic application of techniques for evaluating quality decision making and lack of consensus around a gold standard. This review found 13 techniques that can be used to assess the quality of decision making by pharmaceutical companies, regulatory authorities, and HTA agencies in order to ultimately enable a more consistent and transparent process. Although some of these techniques are scientifically sound and have been developed and tested using robust methodologies, the majority do not possess generalizability to be applied across companies, regulatory authorities, and HTA agencies, and only a proportion have demonstrated practicality and applicability to measure decision making in individuals and organizations against all 10 QDMPs. Indeed, assessing the quality of decision making using a common technique can provide a basis for clear dialogue of issues in decision making within the three stakeholders and ultimately build trust and understanding of what issues are common and which are specific to the three stakeholders. There is also a need to develop for more transparency around how some of the existing techniques and instruments were developed, as well as more testing and routine application for the most promising techniques.

Out of the 13 techniques reviewed, 2, Organizational IQ and QoDoS, have been identified as the most promising, as they conform to all 10 QDMPs. Nevertheless, the Organizational IQ can only be applied to the pharmaceutical industry from an organizational point of view, whereas QoDoS has the potential to capture the issues of companies and agencies alike, as well as evaluating both individuals and their perception of their organizations. This could render QoDoS as the most appropriate measure relative to the other techniques identified. The next steps would be to further test the validity, sensitivity, and reliability of QoDoS across the relevant stakeholders. The overall benefit of systematically assessing the quality of decision making with QoDoS is to enable an increased awareness of biases and best practices but also provide the ability to measure change over time in order to determine the impact of improvement initiatives. Furthermore, such measurements of quality will enable trust, consistency, transparency and timeliness to be built into critical decisions in medicines' development, regulatory review, and HTA.

## Author contributions

MB conceived the study, participated in the development of search and inclusion/exclusion criteria, carried out the primary review of the literature against the inclusion/exclusion criteria, participated in the design/presentation of the flow chart and results tables and drafted the manuscript. NM, SW, and SS participated in the study design, the development of search strategy and inclusion/exclusion criteria, the design/presentation of results tables and helped to draft the manuscript.

## Funding

No funding was received by MB, NM, SW, or SS for the design, collection, analysis, and interpretation of data nor for the writing of the manuscript.

### Conflict of interest statement

The authors declare that the research was conducted in the absence of any commercial or financial relationships that could be construed as a potential conflict of interest. SS and SW have joint copyright of the QoDoS tool.

## Abbreviations

EMA: European Medicines Association
FDA: Food and Drug Administration
HTA: health technology assessment
QDMP: Quality Decision-Making Practices
QoDoS: Quality of Decision-Making Orientation Scheme
R&D: research and development.

## References

[B1] ^*^ Beyer A. R. Fasolo B. de Graeff P. A. Hillege H. L. (2015). Risk attitudes and personality traits predict perceptions of benefits and risks for medicinal products: a field study of European medical assessors. Value Health 18, 91–99. 10.1016/j.jval.2014.10.011 25595239

[B2] ^*^ Blenko M. W. Mankins M. C. Rogers P. (2010). Decide and Deliver: 5 Steps to Breakthrough Performance in Your Organization. Boston, MA: Harvard Business Review Press.

[B3] Buchanan L. O'Connell A. (2006). A brief history of decision making. Harv Bus Rev. 84, 32–41. 16447367

[B4] Bujar M. Donelan R. McAuslane N. Walker S. Salek S. (2016a). Assessing the quality of decision making in the development and regulatory review of medicines: identifying biases and best practices. Ther. Innov. Reg Sci. 51, 250–256. 10.1177/2168479016662681 30231720

[B5] Bujar M. McAuslane N. Salek S. Walker S. (2016b). Quality of regulatory decision-making practices: issues facing companies and agencies. Ther. Innov. Reg. Sci. 50, 487–495. 10.1177/2168479016628573 30227024

[B6] Calnan M. Hashem F. Brown P. (2017). Still elegantly muddling through? NICE and uncertainty in decision making about the rationing of expensive medicines in England. Int. J. Health Ser. [Epub ahead of print]. 10.1177/0020731416689552 28114872

[B7] Canadian Agency for Drugs and Technologies in Health (CADTH) (2012). Common Drug Review Recommendations Options and Deliberative Framework. CDEC. Available at: https://www.cadth.ca/media/cdr/cdr-pdf/CDEC_Deliberative_Framework_e.pdf (Accessed November, 2012).

[B8] Cherny N. I. Sullivan R. Dafni U. (2015). A standardised, generic, validated approach to stratify the magnitude of clinical benefit that can be anticipated from anti-cancer therapies: The European Society for Medical Oncology Magnitude of Clinical Benefit Scale (ESMO-MCBS). Ann. Oncol. 26, 1547–1573. 10.1093/annonc/mdv249 26026162

[B9] Cole A. Marsden G. Devlin N. Grainger D. Lee E. K. Oortwijn W. (2016). New Age Decision Making in HTA: Is It Applicable in Asia? Report of the HTAi 2016 Panel Session, Tokyo, 10–14 May.

[B10] Cook D. Brown D. Alexander R. March R. Morgan P. Satterthwaite G.. (2014). Lessons learned from the fate of AstraZeneca's drug pipeline: a five-dimensional framework. Nat. Rev. Drug Discov. 13, 419–431. 10.1038/nrd4309 24833294

[B11] ^*^ Cowlrick I. Hedner T. Wolf R. Olausson M. Klofsten M. (2011). Decision-making in the pharmaceutical industry: analysis of entrepreneurial risk and attitude using uncertain information. R&D Manage. 41, 321–336. 10.1111/j.1467-9310.2011.00649.x

[B12] Dean J. W. Jr. Sharfman M. P. (1996). Does decision process matter? a study of strategic decision making effectiveness. Acad. Manag. J. 39, 368–396. 10.2307/256784

[B13] Donelan R. Walker S. Salek S. (2015). Factors influencing quality decision making: regulatory and pharmaceutical industry perspectives. Pharmacoepidemiol. Drug Saf. 24, 319–328. 10.1002/pds.3752 25628072

[B14] ^*^ Donelan R. Walker S. Salek S. (2016). The development and validation of a generic instrument, QoDoS, for assessing the quality of decision making. Front Pharmacol. 7:180 10.3389/fphar.2016.00180 27468267 PMC4942854

[B15] Dowding D. Thompson C. (2003). Measuring the quality of judgement and decision-making in nursing. J. Adv. Nurs. 44, 49–57. 10.1046/j.1365-2648.2003.02770.x 12956669

[B16] EUnetHTA. (2016). HTA Core Model. Available online at: https://meka.thl.fi/htacore/ViewHandbook.aspx (Accessed July 8, 2016).

[B17] European Medicines Agency (EMA) (2007). CHMP Rules of Procedure. Available online at: http://www.ema.europa.eu/docs/en_GB/document_library/Other/2009/10/WC500004628.pdf

[B18] European Medicines Agency (EMA) (2011). Benefit-Risk Methodology Project. Work package 3 report: Field tests. Available online at: http://www.ema.europa.eu/docs/en_GB/document_library/Report/2011/09/WC500112088.pdf

[B19] European Medicines Agency (EMA) (2016). Best Practice Guidance for the Parallel Regulatory - HTA Scientific Advice Procedure. Available online at: http://www.ema.europa.eu/docs/en_GB/document_library/Regulatory_and_procedural_guideline/2016/03/WC500203944.pdf

[B20] ^*^ Fischer K. E. Leidl R. Rogowski W. H. (2011). A structured tool to analyse coverage decisions: development and feasibility test in the field of cancer screening and prevention. Health Policy 101, 290–299. 10.1016/j.healthpol.2011.03.002 21529980

[B21] Food and Drug Administration (FDA) (2008). Guidance for FDA Advisory Committee Members and FDA Staff: Voting Procedures for Advisory Committee Meetings. Available online at http://www.fda.gov/downloads/RegulatoryInformation/Guidances/UCM125641.pdf

[B22] Food and Drug Administration (FDA) (2013). Structured Approach to Benefit-Risk Assessment in Drug Regulatory Decision-Making; Draft PDUFA V Implementation Plan. Available online at http://www.fda.gov/downloads/ForIndustry/UserFees/PrescriptionDrugUserFee/UCM329758.pdf

[B23] ^*^ Garbuio M. Lovallo D. Sibony O. (2015). Evidence doesn't argue for itself: the value of disinterested dialogue in strategic decision-making. Long Range Plan. 48, 361–380. 10.1016/j.lrp.2015.09.002

[B24] Goldberg L. R. (1999 March 17). IPIP. Available online at: http://ipip.ori.org (Accessed November 11, 2010).

[B25] Goldberg L. R. Johnson J. A. Eber H. W. Hogan R. Ashton M. C. Cloninger C. R. (2006). The international personality item pool and the future of public domain personality measures. J. Res. Pers. 40, 84–96. 10.1016/j.jrp.2005.08.007

[B26] Guo J. J. Pandey S. Doyle J. Bian B. Lis Y. Raisch D. W. (2010). A review of the quantitative risk-benefit methodologies for assessing drug safety and efficacy—report of the ISPOR risk–benefit management working group. Value Health 13, 657–666. 10.1111/j.1524-4733.2010.00725.x 20412543

[B27] Hammond K. Keeney R. Raiffa H. (1999). Smart Choices: A Practical Guide to Making Better Decisions. New York, NY: Harvard Business School.

[B28] Hassanzadeh S. Gourc D. Marmier F. Bougaret S. (2011). Decision-making in R&D projects, a framework based on fuzzy logic, in International Conference on Production Research (Stuttgart).

[B29] Jekunen A. (2014). Decision-making in product portfolios of pharmaceutical research and development – managing streams of innovation in highly regulated markets. Drug Des. Devel. Ther. 8, 2009–2016. 10.2147/DDDT.S68579 25364229 PMC4211845

[B30] Kahneman D. (2011). Thinking Fast and Slow. London: Penguin Books.

[B31] Leong J. Walker S. Salek S. (2015). A practical approach to communicating benefit-risk decisions of medicines to stakeholders. Front Pharmacol. 6:99. 10.3389/fphar.2015.00099 26124720 PMC4463867

[B32] Liberti L. McAuslane N. Patel P. Breckenridge A. Eichler H. G. Peterson R. (2013). Regulatory review: how do agencies ensure the quality of decision making? Clin. Pharmacol. Ther. 94, 305–308. 10.1038/clpt.2013.127 23963218

[B33] Lovallo D. Sibony O. (2010). The Case of Behavioral Strategy. McKinsey Quarterly. March 2010. Available online at http://www.mckinsey.com/business-functions/strategy-and-corporate-finance/our-insights/the-case-for-behavioral-strategy (Accessed July 8, 2016).

[B34] ^*^ Marangi M. Cammarata S. M. Pani L. (2014). Insights into the decision making of advisory groups to the Italian Medicines Agency. Ther. Innov. Reg. Sci. 48, 696–701. 10.1177/2168479014529571 30227473

[B35] ^*^ Matheson D. Matheson J. (1998). The Smart Organization: Creating value through Strategic R&D. Boston, MA: Harvard Business Review Press.

[B36] Matheson D. Matheson J. (2011). Smart organizations perform better. Res. Technol. Manage. 44, 49–54.

[B37] McAuslane N. Liberti L. Connelly P. (2016a). Real-World Data to Real-World Evidence for Assessing Efficacy and Effectiveness: Opportunities and Challenges for New Medicines Development, Regulatory Review and Health Technology Assessment. Workshop Report. Centre for Innovation In Regulatory Science. Available online at: http://www.cirsci.org/wp-content/uploads/2017/02/CIRS-June-2016-Workshop-Synopsis_6Sept2016.pdf

[B38] McAuslane N. Wang T. Liberti L. Connelly P. (2016b). Commonality in Evidentiary Requirements across Regulatory and HTA Stakeholders. Workshop Report. Centre for Innovation In Regulatory Science. Available online at: http://www.cirsci.org/wp-content/uploads/2017/02/CIRS-September-2016-Workshop-Synopsis_12Dec2016.pdf

[B39] McDowell I. (2006). Measuring Health: A Guide to Rating Scales and Questionnaires. New York, NY: Oxford University Press.

[B40] ^*^ McIntyre T. D. Pappas M. DiBiasi J. J. (2012). How FDA Advisory Committee Members prepare and what influences them. Drug Inform. J. 47, 32–40. 10.1177/0092861512458096 30227490

[B41] ^*^ Mindtools (2013). Decision-Making [Online]. Available online at: https://www.mindtools.com/pages/article/newTED_79.htm (Accessed July 8, 2016).

[B42] Morton A. Airoldi M. Phillips L. D. (2009). Nuclear risk management on stage: a decision analysis perspective on the UK's committee on radioactive waste management. Risk Anal. 29, 764–779. 10.1111/j.1539-6924.2008.01192.x 19178656

[B43] ^*^ Open University (2013). Making Decisions. Available online at: http://www.open.edu/openlearn/ocw/pluginfile.php/58757/mod_oucontent/oucontent_download/epub/83f9b136c23c9909cf9191212ebf0098eb118ebb/making_decisions.epub (Accessed July 8, 2016).

[B44] Pignatti F. Ashby D. Brass E. P. Eichler H.-G. Frey P. Hillege H. L.. (2015). Structured frameworks to increase the transparency of the assessment of benefits and risks of medicines: current status and possible future directions. Clin. Pharmacol. Ther. 98, 522–533. 10.1002/cpt.203 26261064

[B45] Ratliff A. Angell M. Dow R. W. Kuppermann M. Nease R. Fisher R.. (1999). What is a good decision? Effect. Clin. Pract. 2, 185–197. 10539545

[B46] Rogowski W. H. Hartz S. C. John J. H. (2008). Clearing up the hazy road from bench to bedside: a framework for integrating the fourth hurdle into translational medicine. BMC Health Serv. Res. 8:194. 10.1186/1472-6963-8-194 18816378 PMC2569930

[B47] ^*^ Salek S. Mallia-Milanes A. McAuslane N. Walker S. (2012). Development and application of scorecards to assess the quality of a regulatory submission and its review. Ther. Innov. Reg. Sci. 46, 73–83. 10.1177/0092861511427694

[B48] Schnipper L. E. Davidson N. E. Wollins D. S. Tyne C. Blayney D. W. Blum D.. (2015). American Society of Clinical Oncology Statement: A conceptual framework to assess the value of cancer treatment options. J. Clin. Oncol. 33, 2563–2577. 10.1200/JCO.2015.61.6706 26101248 PMC5015427

[B49] Scott S. G. Bruce R. A. (1995). Decision-making style: the development and assessment of a new measure. Educ. Psychol. Meas. 55, 818–831.

[B50] Sharpe P. Keelin T. (1998). How Smithkline Beecham makes better resource-allocation decisions. Harvard Business Rev. 76, 45–57. 10177866

[B51] Strategic Decision Group (SDG) (2011). Decision Quality Webinar: The Art and Science of Good Decision Making. University of Stanford.

[B52] Streiner D. L. Norman G. Cairney J. (2015). Health Measurement Scales: A Practical Guide to Their Development and Use, 5th Edn. Oxford: Oxford University Press.

[B53] Tafuri G. (2013). Exploring the Regulatory Decision-making Process for Medicines. Ph.D. thesis, University of Utrecht.

[B54] Thaler R. H. Sustein C. R. (2009). Nudge: Improving Decisions About Health, Wealth and Happiness. New Haven, CT: Yale University Press.

[B55] Wagner W. (2013). Science in the Administrative Process: A Study of Agency Decisionmaking Approaches. University of Texas School of Law. Available online at: https://www.acus.gov/sites/default/files/documents/Science%20in%20Regulation_Final%20Report_2_18_13_0.pdf (Accessed July 8, 2016).

[B56] Westley F. R. (1990). Middle managers and strategy: microdynamics of inclusion. Strateg. Manage. J. 11, 337–351.

[B57] Wood N. L. (2012). Individual Differences in Decision-Making Styles as Predictors of Good Decision Making. Master's thesis, Graduate College of Bowling Green State University, Ohio.

[B58] World Health Organization (WHO) (2015). Good Review Practices: Guidelines for National and Regional Regulatory Authorities. WHO Technical Report Series, No. 992, Annex 9. Geneva: WHO.

