# Supplementary material for: Evaluating Quality of Decision-Making Processes in Medicines' Development, Regulatory Review, and Health Technology Assessment: A Systematic Review of the Literature
Source: Front Pharmacol. 2017 Apr 10;8:189. doi: 10.3389/fphar.2017.00189 (PMC5385334; doi:10.3389/fphar.2017.00189)
Supplement: Supplementary file 1 [file DataSheet1.docx]

**Appendix**

**The Quality of Decision Making Orientation Scheme (QoDoS) ©**

**Part I: Organisational-level influences**

|  | **Not at all** | **Sometimes** | **Frequently** | **Often** | **Always** |
| --- | --- | --- | --- | --- | --- |
| **A. Decision-Making Approach** | | | | | |
| 1. My organisation evaluates the impact of the decisions it makes |  |  |  |  |  |
| 1. My organisation’s decision making is transparent |  |  |  |  |  |
| 1. My organisation’s decision making is consistent |  |  |  |  |  |
| 1. My organisation uses a structured approach in its decision making |  |  |  |  |  |
| 1. My organisation’s decision making is influenced by external stakeholder’s demands |  |  |  |  |  |
| 1. My organisation qualifies the probability of success in its decision making |  |  |  |  |  |
| 1. My organisation quantifies the probability of success in its decision making |  |  |  |  |  |
| 1. My organisation is open to using better alternatives in its decision making |  |  |  |  |  |
| 1. My organisation encourages innovative decision making |  |  |  |  |  |
| 1. My organisation considers uncertainties in relation to its decision making |  |  |  |  |  |
| 1. My organisation provides training in the science of decision making |  |  |  |  |  |
| 1. My organisation re-examines its decision making as new information becomes available |  |  |  |  |  |
| **B. Decision-making culture** | | | | | |
| 1. My organisation has suffered a negative outcome due to slow decision making |  |  |  |  |  |
| 1. My organisation’s culture has resulted in its inability to make a decision |  |  |  |  |  |
| 1. My organisation’s decision making is influenced by company politics |  |  |  |  |  |
| 1. My organisation’s decision making, it makes the same mistakes as in the past. |  |  |  |  |  |
| 1. My organisation’s decision making its influenced by the vested interest of individuals |  |  |  |  |  |
| 1. My organisation underestimates problems which adversely impacts its own decisions |  |  |  |  |  |
| 1. My organisation continues with projects which should be terminated at an earlier stage |  |  |  |  |  |
| 1. My organisation decision making its influenced by competitors |  |  |  |  |  |
| 1. My organisation’s decision making is influenced by incentives or penalty payments |  |  |  |  |  |
| 1. My organisation effectively communicates the decisions it makes |  |  |  |  |  |
| 1. My organisation provides clear and unambiguous instructions for decision making |  |  |  |  |  |

**Part II: Individual-level influences**

|  | **Not at all** | **Sometimes** | **Frequently** | **Often** | **Always** |
| --- | --- | --- | --- | --- | --- |
| **A. Decision-making competence** | | | | | |
| 1. My decision making is knowledge based |  |  |  |  |  |
| 1. My decision making is consistent |  |  |  |  |  |
| 1. I consider uncertainty and unknowns in my decision-making approach |  |  |  |  |  |
| 1. I generate a SWOT analysis in my decision making |  |  |  |  |  |
| 1. I present contingencies or achievable options as part of my decision making |  |  |  |  |  |
| 1. My decision making is transparent |  |  |  |  |  |
| 1. I understand the context of the decision I am being asked to make |  |  |  |  |  |
| 1. I understand the importance of the decisions I make |  |  |  |  |  |
| 1. I use a structured approach in my decision making |  |  |  |  |  |
| 1. I qualify the probability of success in my decision making |  |  |  |  |  |
| 1. I quantify the probability of success in my decision making |  |  |  |  |  |
| 1. I receive training in the science of decision making |  |  |  |  |  |
| 1. I use intuition or “gut-feeling” in my decision making |  |  |  |  |  |
| 1. My professional experience is important when having to make challenging decisions |  |  |  |  |  |
| **B. Decision-making style** | | | | | |
| 1. Emotion is part of my decision making |  |  |  |  |  |
| 1. I have experienced “paralysis by analysis” caused by my slow decision making |  |  |  |  |  |
| 1. I have experienced a negative outcome by a decision not being made |  |  |  |  |  |
| 1. In my decision making, I make the same mistakes as in the past |  |  |  |  |  |
| 1. Recent or dramatic events greatly impact my decision making |  |  |  |  |  |
| 1. My procrastination has resulted in a negative outcome |  |  |  |  |  |
| 1. My decision making could be improved by assigning weights |  |  |  |  |  |
| 1. I underestimate problems which adversely impact my decision making |  |  |  |  |  |
| 1. I continue with projects which should be terminated at an early stage |  |  |  |  |  |
| 1. I feel that I could make better quality decisions |  |  |  |  |  |

**COPYRIGHT. This questionnaire should not be reproduced without the permission of M.S. Salek** [**m.s.salek@herts.ac.uk**](mailto:m.s.salek@herts.ac.uk) **and S. Walker** [**swalker@cirsci.org**](mailto:swalker@cirsci.org)**.**
